# Duration of basic and attenuated-psychotic symptoms in individuals at clinical high risk for psychosis: pattern of symptom onset and effects of duration on functioning and cognition

**DOI:** 10.1186/s12888-021-03267-2

**Published:** 2021-07-07

**Authors:** Lorna Staines, Ruchika Gajwani, Joachim Gross, Andrew I. Gumley, Stephen M. Lawrie, Matthias Schwannauer, Frauke Schultze-Lutter, Peter J. Uhlhaas

**Affiliations:** 1 Institute for Neuroscience and Psychology, University of Glasgow, Glasgow, UK; 2 Royal College of Surgeons in Ireland, Dublin, Ireland; 3 Institute of Health and Wellbeing, University of Glasgow, Glasgow, UK; 4 Institute for Biomagnetism and Biosignalanalysis, University of Muenster, Muenster, Germany; 5 Department of Psychiatry, University of Edinburgh, Edinburgh, UK; 6 Department of Clinical Psychology, University of Edinburgh, Edinburgh, UK; 7 Department of Psychiatry and Psychotherapy, Medical Faculty, Heinrich Heine University, Düsseldorf, Germany; 8 Department of Psychology and Mental Health, Faculty of Psychology, Airlangga University, Surabaya, Indonesia; 9 University Hospital of Child and Adolescent Psychiatry and Psychotherapy, University of Bern, Bern, Switzerland; 10 Department of Child and Adolescent Psychiatry, Charité Universitätsmedizin, Berlin, Germany

**Keywords:** Clinical high risk for psychosis, Basic symptoms, Attenuated psychotic symptoms, Duration, Psychosis, Cognition

## Abstract

**Introduction:**

Duration of risk symptoms (DUR) in people at clinical high risk for psychosis (CHR-P) has been related to poorer clinical outcomes, such as reduced functioning, but it is currently unclear how different symptoms emerge as well as their link with cognitive deficits. To address these questions, we examined the duration of basic symptoms (BS) and attenuated psychotic symptoms (APS) in a sample of CHR-P participants to test the hypothesis that BS precede the manifestation of APS. As a secondary objective, we investigated the relationship between DUR, functioning and neuropsychological deficits.

**Methods:**

Data from 134 CHR-P participants were assessed with the Comprehensive Assessment of At-Risk Mental State and the Schizophrenia Proneness Interview, Adult Version. Global, role and social functioning and neurocognition were assessed and compared to a sample of healthy controls (*n* = 57).

**Results:**

In CHR-P participants who reported both APS and BS, onset of BS and APS was not significantly related. When divided into short and long BS duration (</> 8 years), CHR-P participants with a longer duration of BS showed evidence for an onset of BS preceding APS (*n* = 8, *p* = 0.003). However, in the short BS duration group, APS showed evidence of preceding BS (*n* = 56, *p* = 0.020). Finally, there were no significant effects of DUR on cognition or functioning measures.

**Conclusion:**

The present findings do not support the view that APS constitute a secondary phenomenon to BS. Moreover, our data could also not confirm that DUR has a significant effect on functioning and cognitive deficits. These findings are discussed in the context of current theories regarding emerging psychosis and the importance of DUR.

**Supplementary Information:**

The online version contains supplementary material available at 10.1186/s12888-021-03267-2.

## Introduction

An important assumption of research on the duration of untreated illness (DUI) in psychosis is the hypothesis of a critical period during which, when left untreated, psychotic symptoms lead to poorer clinical outcomes [1]. Studies have so far primarily focused on the duration of untreated psychosis (DUP), i.e., the time between the first occurrence of frank positive psychotic symptoms and the diagnosis of psychosis or initiation of an antipsychotic treatment [1]. DUP is an important concept that has stimulated research in relation to functional and symptomatic outcomes [2], and, as a result, supported the case for early intervention in individuals experiencing first episode psychosis (FEP) [3]. Research on DUI in the psychosis prodrome yielded similar findings, indicating that longer duration of untreated prodromal symptoms in FEP-patients was associated with lower general functioning [4], and increased negative symptoms [5].

More recently, several studies have examined the relationship between the duration of risk symptoms (DUR) in participants that meet clinical high-risk for psychosis (CHR-P) criteria. Emerging evidence has highlighted that subthreshold symptoms in CHR-P participants may confer a similar relationship with clinical and functional outcomes as in FEP [6]. Specifically, studies in CHR-P participants that meet Ultra High-Risk (UHR) criteria have examined the question of whether longer DUR increases the likelihood for transition to psychosis, but findings have been inconclusive [7–9]. In addition, preliminary evidence from clinical samples indicate that poorer global functioning is related to increased DUR [6, 9, 10]. Thus, lower functioning has been linked to longer duration of UHR symptoms [6, 9] as well as attenuated negative symptoms [7]. One study examined the effect of duration of attenuated psychotic symptoms (APS) symptoms on cognition [11] but no effect was demonstrated. Finally, there is currently only one study that has examined of duration of basic symptoms (BS) on functioning [10] while the link with cognition has not been investigated.

CHR-P participants are defined by meeting UHR and/or BS criteria [12, 13]. UHR criteria are defined by the presence of APS as well as by the presence of brief limited intermittent psychotic symptoms (BLIPS), or by a genetic risk of a psychotic disorder and a significant decrease in mental state or functioning, occurring within the preceding 12 months [12]. In contrast, BS-criteria involve self-experienced cognitive and perceptual anomalies that are hypothesized to detect potential psychosis risk during the early stage of development [14]. Importantly, the co-occurrence of APS and BS has been related to higher transition rates to psychosis [15, 16] (but see [17] for different findings). Moreover, the combination of APS and BS has been linked to pronounced functional impairments and elevated psychopathology in CHR-P participants [17, 18].

Models of emerging psychosis have suggested that APS and BLIPS are preceded by BS [14, 19]. BS are assumed to represent the most immediate symptomatic expression of the neurobiological correlates of schizophrenia [19, 20], while APS have been considered to result from poor or inadequate coping with emerging BS [19]. Currently, there is only limited evidence for this temporal relationship . Schultze-Lutter et al. [21] examined the duration of BS and APS in a FEP sample. Only a third of FEP-patients reported an onset of BS before APS, while another third was characterized by the simultaneous emergence of APS and BS. The remaining participants were characterized by an earlier manifestation of APS compared to BS [21]. Education mediated the relationship between BS and APS onset [21] as well as age of onset and sex [19].

The present study attempted to clarify the relationship between the duration of BS and APS in a sample of CHR-P participants by comparing DUR obtained in routine clinical ratings. Based on previous theoretical evidence [19, 21, 22], we hypothesized that in the majority of CHR-P participants, BS would precede the manifestation of APS. In addition, we investigated the role of DUR on functioning and cognition, given the importance of DUP for clinical outcomes in FEP-patients and emerging evidence for a similar relationship between DUR and functioning in CHR-P participants [6, 7, 9, 11]. Specifically, we hypothesized that longer DUR would impact on functioning as well as cognition.

## Methods

### Recruitment and participants

The data were collected as part of the Youth Mental Health Risk and Resilience (YouR) study [23], a longitudinal study to identify the psychological and neurobiological mechanisms and predictors of psychosis-risk. The YouR-study was approved by the NHS Research Ethical Committee Glasgow and Greater Clyde, and is funded by the Medical Research Council. The majority of the CHR-P participants were recruited from the community through on online detection approach as described previously [24]. Participants provided written informed consent.

To establish CHR-P criteria, the Comprehensive Assessment of At Risk Mental States (CAARMS) [12] and the Schizophrenia Proneness Instrument, Adult version (SPI-A) [13] were used. Interviews were administered by trained research assistants, MSc and PhD level-researchers. Inter-rater reliability (IRR) of CHR-P status as determined by the CAARMS and SPI-A ratings was good to excellent (CAARMS: 92.0%; SPI-A: 95.7%). CAARMS criteria for CHR-P were as follows: 1) APS group (subthreshold psychotic syndrome present in the last year) 2) BLIPS and 3) Genetic risk and functional decline (GRFD). SPI-A criteria included COPER and COGDIS. CHR-P participants were excluded for current or past diagnosis of an axis-I psychotic disorder.

In addition, a control group (*n* = 60, 42 female, 18 male) was recruited without an axis I diagnosis or family history of psychotic disorders. Potential participants were screened with the Mini-International Neuropsychiatric Interview (MINI [25]). and completed the demographic, functional and neurocognitive measures.

### Assessment of DUR

Duration of APS and BS was obtained from CAARMS and SPI-A assessments. CAARMS items from the positive scale were considered if a) a frequency score of 3–5 and b) an intensity rating of ≥3 on the unusual thought content or non-bizarre ideas, or perceptual abnormalities scale or ≥ 4 on the disorganised speech scale was met [12]. BS items were included when symptoms had a frequency rating of > 3. APS and BS with symptom duration since early childhood and no change in symptom severity were excluded as these are considered more equivalent to schizotypal traits.

Only DUR-scores for which the participant was able to identify the month of onset, the year of onset (treated as January 1st of that year), or age when the symptom emerged, were included for analysis. DUR was determined by the number of months from symptom onset – irrespective of the initial frequency of occurrence – until the date of the baseline interview, in line with previous studies [6–9, 11, 21].

### Clinical and cognitive assessments

In addition to the CAARMS and SPI-A interviews, all participants were administered the M.I.N.I [25]. Functioning was assessed with the global assessment of functioning (GAF) and the social and role scales (GF: social and role) [26] at baseline. In addition, the Brief Assessment of Cognition in Schizophrenia Battery (BACS) [27]) as well as the following tasks from the University of Pennsylvania Computerized Neuropsychological Testing Battery (PennCNP [28]): a) Continuous Performance Test b) the N-Back Task, and c) Emotion Identification Task, were administered.

### Statistical analysis

All statistical analysis were performed using R [29]. Criteria-relevant CAARMS and SPI-A symptoms with the longest reported duration for each participant were entered into the analysis. For individuals who reported both APS and BS symptoms, the symptom with the longest duration was entered into the analysis.

Group differences in clinical and demographic measures were calculated using Pearson’s Chi-squared test, Bartlett’s test of homogeneity of variances, Kruskal-Wallis rank sum test, and one-way ANOVA tests. To assess the relationship between onset of BS and APS, duration of APS and BS were separately recorded and z-scored using the means and standard deviations in the CHR-P group. Paired t-tests and robust linear regression was used to evaluate the relationship between APS and BS. Secondary analyses were conducted by dividing CHR-P participants with short vs. long duration of BS [22] which were defined as </> 1 SD from the mean. Cohen’s d was calculated to measure effect sizes.

For analysis of APS/BS duration and functioning measures, quantile regressions were used to examine the relationship with DUR, as GAF scores and social and role functioning failed to meet the assumptions of parametric measures. In addition, neurocognitive data was converted into standardized z-scores for each cognitive domain by using the means and standard deviations of the control group in line with previous studies [6, 9]. Linear regression was used to examine the relationship between DUR and cognition.

## Results

### Demographic and cognitive data

The CHR-P participants differed from controls in age at baseline and years of education. Specifically, the CHR-P group had poorer functioning in global, role and social measures (Table 1).

**Table 1:** Demographic, functional and clinical characteristics of CHR-P and Controls

	Controls	CHR-P	df	H/χ^2^	p
**Number of participants**	60	134			
**Female participants, n (%)**	42 (68.9)	93 (69.4)	1	χ^2^ = 0.00	1.0
**Age at baseline, median (range)**	22 (18–32)	20 (16–34)	1	H = 6	**0.01**
**Age at onset, median (range)**	–	17 (4–31)			
**Years in education, median (range)**	16 (12–24)	15 (8–26)	1	H = 127	**<0.001**
**UK citizen, N (%)**	28 (45.9)	97 (72.4)	1	χ^2^ = 12	**<0.001**
**GAF, median (range)**	88 (67–97)	58 (21–95)	1	H = 32	**<0.001**
**GF: Role, median (range)**	9 (5–9)	8 (4–9)	1	H = 55	**<0.001**
**GF: Social, median (range)**	9 (7–9)	8 (5–9)	1	H = 67	**<0.001**
**Current medication, n (%)**					
No medication	60 (98.36)	68 (50.75)	1	χ^2^ = 42	**<0.001**
Anti-psychotic	0	2 (01.49)	1	χ^2^ = 0.01	0.9
Mood stabiliser	0	1 (0.75)	1	χ^2^ = 0.00	1.0
Anti-depressant	0	30 (22.39)	1	χ^2^ = 15	**<0.001**
Anti-convulsant	0	1 (0.75)	1	χ^2^ = 0.00	1.0
Other	1 (1.64)	13 (9.70)	1	χ^2^ = 3	0.06
Multiple	0	21 (15.67)	1	χ^2^ = 8	**0.005**
**Lifetime diagnosis, n (%)**					
No diagnosis	57 (95.0)	21 (15.67)	1	χ^2^ = 126	**<0.001**
Anxiety disorder	2 (3.33)	89 (66.42)	1	χ^2^ = 77	**<0.001**
Mood disorders	0	86 (64.18)	1	χ^2^ = 73	**<0.001**
Eating disorders	0	13 (9.70)	1	χ^2^ = 5	**0.03**
Alcohol dependence/abuse	1 (1.67)	40 (29.85)	1	χ^2^ = 18	**<0.001**
Substance dependence/abuse	0	21 (15.67)	1	χ^2^ = 10	**0.001**
Suicidality n (%)					
Suicidality	1 (1.67)	72 (53.73)	1	χ^2^ = 49	**<0.001**
No diagnosis	59 (98.33)	62 (46.27)			

*Abbreviations:* *CHR-P* clinical high risk for psychosis, *df* degrees of freedom, *H* Kruskal-Wallis H test, χ^2^ Chi-Squared test, *GAF* Global Assessment of Functioning, *GF:Role/Social* Global Functioning Role & Social Scale

For 26 CHR-P participants, no cognitive data was available. For the remaining CHR-Ps, there were significant impairments compared to controls in working memory, executive function, attention accuracy and in the BACS composite score. In addition, CHR-P participants were characterized by slower reaction times in the emotion recognition task (Table 2).

**Table 2:** Cognition in CHR-P

	Controls (***n*** = 57)		CHR-P (***n*** = 108)		df	F	(95%) CI	p	Cohen’s d
	M	SD	M	SD					
**BACS**									
Verbal memory	0	1	−0.23	1.22	56	1	0.92, 2.23	0.10	−0.2
Working memory	0	1	− 0.07	1.26	56	2	0.99, 2.41	**0.053**	− 0.06
Motor speed	0	1	−0.79	1.12	56	1	0.78, 1.89	0.401	−0.73
Attention & processing speed	0	1	−0.49	1.16	56	1	0.84, 2.05	0.20	−0.44
Verbal fluency	0	1	−0.14	0.62	56	0.8	0.49, 1.19	0.20	−0.29
Executive function	0	1	−0.04	1.36	56	2	1.1, 2.8	**0.011**	−0.03
BACS composite score	0	1	−0.64	1.51	56	2	1.4, 3.4	**<0.001**	−0.46
**Penn CNB**									
Emotion recognition accuracy	0	1	−0.16	1.00	56	1	0.62, 1.50	0.900	−0.15
Emotion recognition RT	0	1	0.43	1.28	56	2	1.0, 2.5	**0.030**	0.36
Working memory accuracy	0	1	−0.29	1.15	56	2	1.1, 2.8	**0.010**	−0.3
Working memory RT	0	1	−0.09	0.80	56	0.7	0.41, 1.00	**0.049**	−0.1
Attention accuracy	0	1	−0.37	1.29	56	2	1.0, 2.5	**0.041**	−0.3
Attention RT	0	1	−0.13	0.88	56	1	0.6 1.5	0.80	−0.18

*Abbreviations*: *M* Mean, *SD* Standard deviation, *df* degrees of freedom, *F* F-statistic, *CI* Confidence intervals, *p p*-value, *RT* Reaction time

### CHR-P with APS & BS vs. APS or BS alone

Of the CHR-P participants screened, 77.57% of those with APS reported duration of symptoms (*n* = 109), and 68.67% of those with BS reported duration data (*n* = 89). A total of 134 CHR-P reported APS or BS duration (87.58%).

**Fig. 1:**
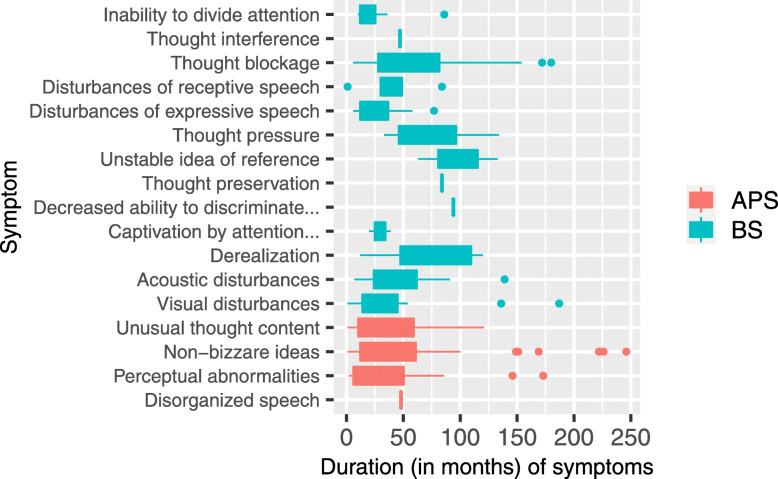
Median duration of first onset APS and/or BS Disturbances of abstract thinking (O3) was not reported by any participant as a first onset basic symptom, so excluded from the figure. Abbreviations: *APS* attenuated psychotic symptom, *BS* basic symptom, *Decreased ability to discriminate …* Decreased ability to discriminate between ideas/perception and fantasy/true memories, *Captivation of attention …* Captivation of attention by details of the visual field

The average duration for APS was 43 months and for BS was 51 months (Table 3), but individual symptoms showed significantly different duration (Fig. 1). CHR-P participants reporting both APS/BS showed a general trend towards longer DUR, compared to those with only APS or BS, but this finding was not significant.

**Table 3:** Demographic and Clinical Characteristics of CHR-P Subgroups

	APS & BS	APS	BS	df	K^2^/H/χ^2^	p
**Number of participants**	64	45	25			
**Female participants, n (%)**	44 (68.8)	34 (75.6)	15 (60.0)	2	χ^2^ = 2	0.4
**Age at baseline, median (range)**	21 (16–34)	19 (16–32)	21 (17–31)	2	H = 4	0.1
**Age at onset, median (range)**	17 (5–29)	18 (4–31)	17 (5–25)	2	H = 2	0.3
**Years in education, median (range)**	15 (10–26)	14 (8–21)	15 (11–18)	2	H = 6	**0.04**
**UK citizen, N (%)**	46 (71.9)	31 (68.9)	20 (80.0)	2	χ^2^ = 0.8	0.7
**ARMS**						
APS criteria, n = 109 (81.34%)	64	45	–	1	χ^2^ = 0.00	1.0
BLIPS criteria	0	0	–			
Severity score, mean (range)	16.31 (1–30)	15.31 (9–25)	–	1	K^2^ = 0.36	0.549
**COPER**						
Criteria, *n* = 88 (61.94%) ^a^	61	–	27		χ^2^ = 1.17	0.280
Sum score, mean (range)	4.07 (3–6)	–	4.05 (3–6)		K^2^ = 2.85	0.092
**COGDIS**						
Criteria, *n* = 57 (32.09%)^a^	39	–	18		χ^2^ = 0.70	0.403
Sum score, mean (range)	4.35 (3–6)	–	4.13 (3–6)		K^2^ = 0.18	0.669
**Mean duration (SD), in months**						
APS	46 (45)	39 (54)	–		F = 0.54	0.46
BS	54 (45)	–	48 (43)		F = 0.38	0.54
**Median duration (range), in months**						
APS	37 (1–226)	12 (2–246)	–		H = 3	0.08
BS	44 (1–180)	–	37 (3–187)		H = 0.6	0.4
**GAF, median (range)**	55 (21–95)	57 (21–86)	62 (34–95)		H = 3	0.2
**GF: Role, median (range)**	8 (4–9)	8 (4–9)	8 (6–9)		H = 2	0.4
**GF: Social, median (range)**	8 (5–9)	8 (5–9)	8 (6–10)		H = 0.8	0.7
**Current medication, n (%)**						
No medication	28 (43.8)	23 (51.1)	12 (48.0)	2	χ^2^ = 0.5	0.8
Anti-psychotic	1 (1.6)	1 (2.2)	0	2	χ^2^ = 0.5	0.8
Mood stabiliser	0	0	1 (4.0)	2	χ^2^ = 4	0.1
Anti-depressant	16 (25.0)	11 (24.4)	3 (12.0)	2	χ^2^ = 2	0.4
Anti-convulsant	0	0	1 (4.0)	2	χ^2^ = 4	0.1
Other	9 (14.1)	3 (6.7)	3 (12.0)	2	χ^2^ = 2	0.5
Multiple	9 (14.1)	7 (15.6)	5 (20.0)	2	χ^2^ = 0.4	0.8
**Lifetime diagnosis, n (%)**						
No diagnosis	5 (7.8)	3 (6.7)	4 (16.0)	2	χ^2^ = 2	0.3
Anxiety disorder	45 (70.3)	32 (71.1)	12 (48.0)	2	χ^2^ = 3	0.2
Mood disorders	57 (89.1)	27 (60.0)	12 (48.0)		χ^2^ = 4	0.1
Eating disorders	6 (9.4)	6 (13.3)	1 (4.0)		χ^2^ = 1	0.5
Alcohol dependence/abuse	20 (31.3)	14 (31.1)	6 (24.0)		χ^2^ = 0.2	0.9
Substance dependence/abuse	7 (10.9)	11 (24.4)	3 (12.0)		χ^2^ = 4	0.2
Suicidality n (%)						
Suicidality	35 (20.0)	24 (45.8)	13 (23.1)		χ^2^ = 0.1	0.9

^a^ = note 37 participants met both COGDIS & COPER criteria. *Abbreviations*: *X*^*2*^ Pearson’s Chi-squared test, *K*^*2*^ Bartlett test of homogeneity of variances, *H* Kruskal-Wallis rank sum test, *F* one way ANOVA, *ARMS* at-risk mental state, *APS* attenuated psychotic symptoms, *BLIPS* brief limited intermittent psychotic symptoms, *BS* Basic symptoms, *COPER* Cognitive-perceptive basic symptoms, *COGDIS* Cognitive disturbances basic symptoms

### Relationship between APS and BS onset

Of the CHR-P participants who met both APS/BS-criteria (*n* = 64), *n* = 31 (48.4%) reported an onset of BS prior to APS onset, while *n* = 24 (37.5%) reported APS onset prior to BS. Nine (14.1%) CHR-P participants reported APS and BS onset occurring in the same month. No significant difference in duration was found for BS onset preceding APS (paired t-test: t = 0.02, df = 63, *p* = 1.0). Robust linear regression analysis which accounted for outliers also failed to find a relationship between BS and APS onset (robust regression: B = − 0.065, SE = 0.129, t-value = − 0.502, F-test =0.252, *p* = 0.616).

Secondary analyses examining the relationship between short or long BS duration (defined as >/< 1 SD (≈ 8 years)) and onset of APS were conducted to examine potential group differences. Short BS duration showed a significant relationship (paired t-test: t = − 2.40, df = 55, *p* = 0.020, MD = − 0.3, 95%CI [− 0.54, − 0.05]; effect size d = − 0.17), indicating that APS preceded BS onset. Robust linear regression failed to find a significant relationship (robust regression: B = 0.227, SE = 0.149, t-value = 1.52, F = 2.27, *p* = 0.138). An opposite pattern was observed, however, for the long BS duration group, with BS occurring prior to APS onset (paired t-test: t = 4, df = 7, *p* = 0.003, MD = 2.1, 95% CI [0.96–3.24]), which persisted when outliers were accounted for (robust regression: B = − 1.318, SE = 0.441, t-value = − 2.988, F = 8.819, *p* = 0.025; effect size d = − 2.8).

Paired t-tests were conducted to determine between-group differences of BS onset compared to APS onset. Education (individuals with/without third level education), age of onset (>/< 18 years of age at onset of either symptom type), and gender were separately analysed. No significant effects were observed that mediated the pattern of onset between BS and APS (Supplemental Table 1).

### Relationship between DUR and functioning

19 CHR-P participants did not complete assessments for functional measures and were excluded from the analysis. Duration of APS and BS showed no significant effects on GAF scores, role functioning or social functioning in any of the CHR-P groups (Table 4).

**Table 4:** Effect of Duration of CHR-P Symptoms on Functional Measures

	B	SE	t	95% CI	p
**APS** (*n* = 45)					
GAF	− 0.033	0.030	−1.11	− 0.06, 0.05	0.272
GF: Role	0.000	0.004	0.00	−0.00, 0.01	1.00
GF: Social	0.000	0.003	0.00	−0.01, 0.01	1.00
**BS** (*n* = 25)					
GAF	−0.16	0.13	−1.22	− 0.38, 0.01	0.241
GF: Role	−0.006	0.005	−1.28	− 0.01, 0.01	0.215
GF: Social	−0.006	0.010	− 0.60	− 0.02, 0.03	0.557
**APS & BS** (n = 64)					
GAF	0.035	0.041	0.87	−0.06, 0.09	0.389
GF: Role	−0.005	0.004	−1.15	−0.01, 0.00	0.256
GF: Social	0.00	0.004	0.00	−0.01, 0.01	1.00

*Abbreviations*: *B* unstandardized estimate, *SE* Standard error, *t* t-value, *CI* Confidence intervals, *p p*-value

### Relationship between DUR and cognition

Longer APS duration was associated with impaired verbal fluency but this effect did not survive corrections for multiple comparisons (*p* = 0.22). No additional cognitive subtest correlated with APS or BS duration (Table 5).

**Table 5:** Association of Duration of CHR-P Symptoms on Cognition

	B	SE	t	95% CI	R^**2**^	p
**APS (*n*** **= 45)**						
BACS						
Verbal memory	0.001	0.004	0.30	−0.01, 0.01	0.002	0.767
Working memory	0.002	0.004	0.57	−0.00, 0.01	0.009	0.571
Motor speed	−0.002	0.004	−0.68	− 0.01, 0.01	0.038	0.503
Processing speed	−0.001	0.003	−0.16	− 0.01, 0.01	0.001	0.876
Verbal fluency	−0.003	0.001	−2.4	−0.01, 0.00	0.13	**0.022**
Executive function	0.006	0.004	1.25	−0.00, 0.01	0.040	0.218
BACS composite score	0.002	0.005	0.47	−0.01, 0.01	0.006	0.643
Penn CNP						
Emotion recognition accuracy	0.003	0.003	1.13	−0.00, 0.01	0.033	0.267
Emotion recognition RT	0.003	0.003	0.86	−0.00, 0.01	0.020	0.396
Attention accuracy	0.004	0.004	0.82	−0.01, 0.01	0.018	0.416
Attention RT	− 0.004	0.002	−1.5	− 0.01, 0.01	0.057	0.143
Working memory accuracy	0.001	0.004	0.22	−0.01, 0.01	0.001	0.826
Working memory RT	0.001	0.002	0.31	−0.00, 0.01	0.003	0.755
**BS (*n*** **= 25)**						
BACS						
Verbal memory	0.008	0.006	1.17	−0.01, 0.02	0.071	0.256
Working memory	0.009	0.007	1.30	−0.01, 0.03	0.086	0.211
Motor speed	−0.01	0.006	−0.90	−0.02, 0.01	0.804	0.382
Processing speed	0.003	0.007	0.39	−0.01, 0.02	0.008	0.701
Verbal fluency	0.004	0.004	0.99	−0.01, 0.01	0.051	0.338
Executive function	−0.001	0.008	−0.07	−0.02, 0.02	0.000	0.938
BACS composite score	0.006	0.010	0.68	−0.01, 0.03	0.025	0.506
Penn CNP						
Emotion recognition accuracy	0.005	0.006	0.93	−0.01, 0.02	0.046	0.366
Emotion recognition RT	−0.002	0.008	−0.24	−0.02, 0.01	0.003	0.815
Attention accuracy	0.003	0.007	0.35	−0.01, 0.02	0.007	0.730
Attention RT	−0.006	0.005	−1.0	−0.02, 0.01	0.056	0.317
Working memory accuracy	−0.001	0.007	−0.12	−0.02, 0.01	0.001	0.902
Working memory RT	−0.004	0.005	−0.87	−0.01, 0.01	0.040	0.396
**APS & BS (*n*** **= 64)**						
BACS						
Verbal memory	−0.002	0.003	−0.64	−0.01, 0.00	0.007	0.523
Working memory	0.001	0.003	0.38	−0.01, 0.01	0.003	0.703
Motor speed	−0.002	0.003	−0.65	−0.01, 0.00	0.007	0.519
Processing speed	−0.002	0.003	−0.76	−0.01, 0.00	0.010	0.452
Verbal fluency	0.000	0.002	0.18	−0.00, 0.00	0.001	0.856
Executive function	−0.006	0.003	−1.95	−0.01, 0.00	0.063	0.056
BACS composite score	−0.004	0.003	−1.23	−0.01, 0.01	0.026	0.244
Penn CNP						
Emotion recognition accuracy	−0.003	0.002	−1.02	−0.01, 0.00	0.018	0.313
Emotion recognition RT	0.000	0.003	0.01	−0.01, 0.01	0.000	0.996
Attention accuracy	−0.003	0.003	−1.1	−0.01, 0.00	0.020	0.282
Attention RT	−0.001	0.002	−0.58	−0.01, 0.00	0.006	0.563
Working memory accuracy	−0.004	0.003	−1.2	−0.01, 0.00	0.026	0.218
Working memory RT	−0.001	0.002	−0.34	−0.01, 0.00	0.002	0.735

*Abbreviations*: *RT* Reaction time, *B* unstandardized estimate, *SE* Standard error, *β* standardized estimate, *t* t-value, *CI* Confidence intervals, *p p*-value

## Discussion

The current study examined BS and APS in a CHR-P sample to test current models of emerging psychosis as well as the effects of DUR on functioning and cognitive deficits. The main finding is that we could not confirm the hypothesis that BS preceded the development of APS. Moreover, we could not replicate findings indicating that longer DUR was related to poor functioning [6, 9, 10], but our findings are consistent with previous evidence suggesting that cognitive deficits were not influenced by DUR [11].

DUR in the current CHR-P sample that was largely community-recruited was longer than in previous CHR-P samples obtained from clinical pathways [6–8, 11]. Accordingly, one possibility is that longer DUR may be the result of the absence of an early intervention service in our catchment area and thus we detected CHR-P participants at a later stage of illness. While the impact of recruitment strategies on the clinical characteristics of CHR-P probands continues to be discussed [30, 31], it is important to note that the CHR-P participants in the current study were characterized by similar neuropsychological and global functioning scores as those reported for clinically-referred CHR-P cohorts [32].

An important finding is that criteria-relevant BS did not precede APS in the majority of CHR-P participants. In fact, APS onset preceded BS in a considerable portion of the sample (37.5%, *n* = 24). In addition, there was no significant evidence between duration of BS and APS. Moreover, in the short BS duration group (< 1 SD ≈ 8 years), APS also preceded BS onset. Only in the small long BS duration group (*n* = 8) was there evidence of BS duration being significantly longer than APS.

Analyses for between group differences based on sex, age at onset, and years of education also failed to find evidence of BS preceding APS onset. This finding differs from data reported in a FEP-cohort, which linked the sequence of BS onset preceding APS to higher school education, male gender and an onset of mental health issues before age 18 [21, 22].

Moreover, in contrast to previous studies [6, 9, 10], we found no significant effect of APS or BS duration on GAF scores, role or social functioning (but see [7]). One possibility is that community recruited CHR-P participants may reflect different trajectories for the development of psychosis than those who are identified through clinical pathways, which may be characterized by a more insidious onset, for example.

Confirming our previous findings [32], CHR-P participants were characterized by moderate impairments in cognition, in particular in regards to working memory, attention and executive processes. In line with Chon and colleagues [11], there was only marginal evidence for a relationship between DUR and cognitive performance in CHR-P participants, however. This finding is also consistent with data in FEP-patients [33], highlighting that DUP does not have an impact on cognitive deficits during early-stage psychosis.

Finally, several studies [15, 16, 18] have indicated that CHR-P participants with a combination of APS and BS had a higher risk of transition to psychosis as well as more severe psychopathology and lower functioning [16, 17], raising the possibility that this subgroup of CHR-P participants may also be characterized by differences in DUR. Again, we could not confirm this hypothesis, although a moderate trend was observable for a longer DUR in CHR-P individuals with both APS and BS. Similarly, functioning was also not significantly lower in those with APS and BS compared to those with only APS or BS, in contrast to previous evidence [18].

### Limitations

There are several limitations to the current findings. Firstly, as in previous studies, DUR relied on retrospective self-reports, which are potentially vulnerable to both recognition and recall bias [21, 22]. Moreover, the overall sample size as well as the number of participants who reported both APS and BS symptoms were modest, yet similar to previous findings [21]. The current sample was primarily collected from a community sample and previous studies have found differences in symptom severity and functioning scores between community and help-seeking CHR-P participants [34] as well as lower transition rates [31].

## Conclusions

There is currently only limited evidence on the duration of CHR-P symptoms, the relationship between BS and APS, and their relationship to functional outcomes and cognition. One key finding of this paper is that APS does not emerge as a secondary consequence of BS. Indeed, in the short BS duration group, evidence showed APS preceded BS. Importantly, the current findings also highlight that community-recruited CHR-P participants experienced substantially longer duration of subthreshold symptoms compared to previous studies. However, we could not confirm that DUR had a significant effect on functioning or cognition. This raises the question whether DUR is an important variable for the understanding of emerging psychosis in CHR-P participants or a suitable potential target for early intervention.

To address these question, further studies in larger CHR-P samples need to be conducted that utilize more sophisticated assessment of DUR, including development of specific tools and guidelines. This might offer greater clarity into the potential link between DUR on clinical and functional outcomes in CHR-P participants and for measures to reduce DUR in emerging psychosis.

## Supplementary Information

**Additional file 1:** **Supplementary Table 1.** Summary of between group differences of onset of basic symptoms compared to onset of attenuated psychotic symptoms.

## Data Availability

The datasets generated during and/or analysed during the current study are available from the corresponding author on reasonable request.
