# Supplementary material for: Duration of basic and attenuated-psychotic symptoms in individuals at clinical high risk for psychosis: pattern of symptom onset and effects of duration on functioning and cognition
Source: BMC Psychiatry. 2021 Jul 7;21:339. doi: 10.1186/s12888-021-03267-2 (PMC8265048; doi:10.1186/s12888-021-03267-2)
Supplement: Supplementary file 1 — Additional file 1: Supplementary Table 1. Summary of between group differences of onset of basic symptoms compared to onset of attenuated psychotic symptoms. [file 12888_2021_3267_MOESM1_ESM.pdf]

Supplementary table 1: Summary of between group differences of onset of basic symptoms compared to onset of attenuated psychotic symptoms

	**Paired t-test**							**Robust regression**				
	**N**	**df**	**t**	**MD**	**p**	**95% CI**	**Cohen's d**	**B**	**SE**	**t-value**	**F**	**p**
Male only	19	18	-0.01	-0.004	1.00	-0.69, 0.68	0.00	-0.055	0.188	-0.293	0.09	0.770
Female only	45	44	0.04	0.007	1.00	-0.36, 0.37	-0.01	0.094	0.093	1.021	1.02	0.319
< 18 years	41	40	0.1	0.02	0.80	-0.4, 0.44	0.15	-0.015	0.136	-0.113	0.01	0.911
> 18 years	23	22	-0.1	-0.026	0.90	-0.53, 0.48	-0.10	-0.016x	0.085	-0.193	0.04	0.847
No 3rd level education	9	7	0.4	0.18	0.70	-0.78, 1.14	-0.22	-0.037	0.195	-0.191	0.04	0.855
3rd level education	55	54	-0.1	-0.021	0.90	-0.37, 0.33	0.02	0.068	0.094	0.722	0.5	0.472
